# Bone remodeling simulation using spatial influence function in macroscopic cube case

**DOI:** 10.3389/fbioe.2024.1498812

**Published:** 2024-11-29

**Authors:** Isna Riski Safira, Martin Ramette, Spyros D. Masouros, Anthony M. J. Bull

**Affiliations:** Department of Bioengineering, Imperial College London, London, United Kingdom

**Keywords:** bone remodeling, spatial influence function, 3D simulation, parameter sensitivity, finite element analysis

## Abstract

Bone has the capability to adapt its density in response to mechanical stimuli through a process known as bone remodeling, which has been simulated *in silico* using various algorithms in several studies, with Strain Energy Density (SED) being a commonly used driving parameter. A spatial influence function has been introduced in addition to the remodeling algorithm, which accounts for the influence of neighboring regions on local mechanical stimuli, thereby reducing artificial mesh dependency and mimicking cellular communication in bone. However, no study has implemented the SED-driven algorithm with spatial influence function on a macroscopic 3D bone structure, and there is no physiological explanation on the value used in remodeling parameter. The goal of this study was to assess the effect of the spatial influence function’s parameters on the resulting 3D simple cubic structure under compressive loading through a sensitivity analysis. The results demonstrated that the spatial influence function enabled the density distribution to propagate in directions not only aligned with external loads, thus simulating the work of cellular communication. This study also underscores the importance of selecting appropriate parameter values to accurately reflect physiological conditions in bone remodeling simulations, since different parameters influence not only bone mineral density but also the architecture of the resulting bone structure. This work represents a step forward in understanding the interplay between mechanical stimuli and bone remodeling in three dimensions, providing insights that could improve the accuracy of computational models in simulating physiology and pathophysiology.

## Introduction

Bone has the self-optimizing capabilities to control its mass, density, and structure in direct response to the mechanical stimuli exerted onto it, in a process known as bone remodeling (Frost, 1994). Bone remodeling can be accomplished through bone formation by osteoblasts and bone resorption by osteoclasts, and it is mediated by osteocytes, which are thought to function as “sensing cells”. Various *in silico* bone remodeling algorithms have been proposed to simulate the relationship between density changes and the amount of stimulus induced by mechanical loading, using variables such as effective stress (Doblare and Garcia, 2002), strain (Turner et al., 1997), microdamage (Carter et al., 1989), and Strain Energy Density (SED) (Huiskes et al., 1987), representing the effect of osteoblasts, osteoclasts, osteocytes and the interplay between them. SED is the most commonly chosen driving parameter for bone-remodeling algorithms in the literature (Giorgio et al., 2019; Gong et al., 2013; Lian et al., 2010; Mullender et al., 1994; Weinans et al., 1992), due to (a) its ability to capture the integral measure (magnitude and distribution) of the two main mechanical stimuli that regulate load-induced bone adaptation (Rosa et al., 2015): mechanical strain from cell deformation and stress from fluid flow), (b) its high correlation with osteoblastic and osteoclastic activity (Webster et al., 2015), and (c) its capacity to allow density adaptation in response to functional requirements (Andreaus et al., 2014; Carter et al., 1989). Given a specific mechanical scenario, the SED can be estimated and input into the remodeling algorithm through the use of finite element (FE) analysis. The ability of FE analysis to accurately simulate a wide range of *in vivo* scenarios of healthy and abnormal bone structures is well documented.

The spatial influence function was introduced by Mullender et al. (1994) to reduce the discontinuous density distribution found in previous FE simulation (Weinans et al., 1992). This function effectively means that the mechanical stimulus experienced by a region is also influenced by the one of the neighboring regions, simultaneously attenuating the artificial mesh dependency induced by the FE analysis (Sigmund and Petersson, 1998) and mimicking a form of cellular communication akin to the role of osteocytes *in vivo* (Schaffler et al., 2014). Furthermore, bone cell signaling, facilitated by soluble chemokines, has been shown to enable non-local bone remodeling regulation (Khosla, 2001). The spatial influence function in bone remodeling simulation has been used in several studies (Kumar et al., 2011; Ruimerman et al., 2005; Tsubota and Adachi, 2006; Zhang et al., 2023) and has demonstrated a stabilizing effect in dynamic spatial contexts compared to bone remodeling simulations without the spatial influence function (Ryser and Murgas, 2017). The impact of this spatial influence function on computational bone remodeling algorithms is the specific focus of the present study.

This SED-driven algorithm with the spatial influence function has been previously implemented in simplified 2D geometries (Jang et al., 2009; Lian et al., 2010; Mullender et al., 1994; Rosenberg and Bull, 2018). Although 2D FE models are computationally efficient, their simplification lies not only in geometry but also in loading, being incapable of simulating bending and torsional loads. The spatial influence function has been previously applied in 3D trabecular meshes with a porous voxel microstructure (Gerhard et al., 2009; Ruimerman et al., 2005; Tsubota and Adachi, 2006), which has voxel size smaller than the trabecular thickness. However, this approach may limit the potential for simulating new trabecular bone growth in empty spaces, making it more difficult to represent accurately communication within the osteocytic network. Validating microstructure FE models against experimental data is challenging due to the lack of comprehensive *in vivo* measurements at the microscale and the difficulty in replicating complex physiological conditions in laboratory settings. No studies in the literature have implemented the SED-driven algorithm with spatial influence function on a macroscopic 3D bone structure; a continuous solid volume approach rather than modeling individual trabeculae; and so it is still unknown if the parameter values used in 2D applications can effectively recreate trabecular structures in 3D applications. Using a macroscopic 3D bone model, the application can be scaled up to larger domains, enabling more efficient computational time and the incorporation of more physiological boundary conditions.

The aim of this study was to implement an SED-driven remodeling algorithm, with a spatial influence function into a simple 3D geometry, specifically a cube, and to run a sensitivity analysis to quantify the effect of the function’s parameters under multiaxial loading on the resulting 3D-bone structure. This analysis enables the quantification of the dependency between the function’s parameters and bone density, helping to choose the appropriate parameters for the application of interest.

## Methods

**FIGURE 1:**
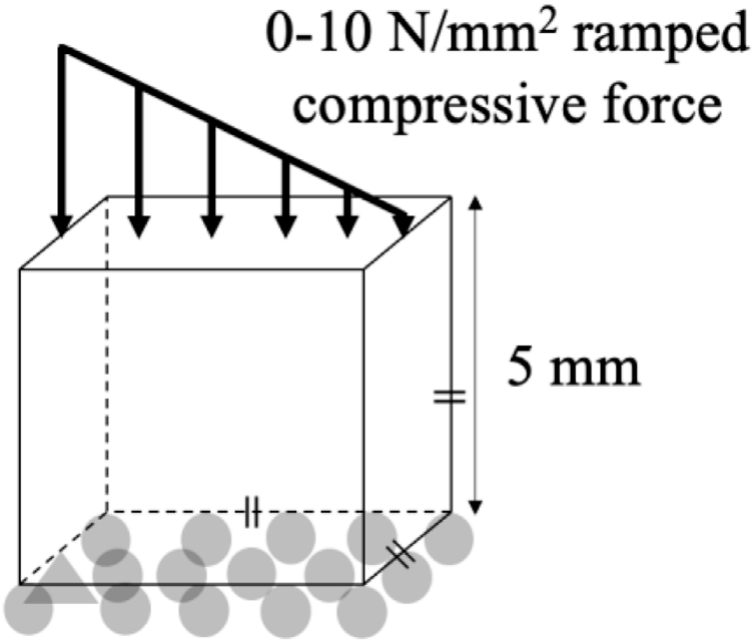
Boundary conditions of the cube.

An FE model of a simple, reference geometry comprising a 5 × 5 × 5 mm cube was developed in Marc Mentat (2022.4, MSC Software, United States). The model comprised 8,000 identical cubic elements, 0.25 mm in edge length (Figure 1). A bone remodeling algorithm was implemented in Fortran, in which the relationship between the SED, the spatial influence function, and the density rate of change was governed by Equation 1.

$$\frac{d\rho \left(x\right)}{dt}=\tau \sum_{j=1}^{N}f_{j}\left(x\right)\left(\frac{U_{a,j}}{\rho_{j}}-k\right)$$ (1)

where *ρ* is a finite element’s density, k is a constant reference value corresponding to homeostasis, τ is a fixed time constant, *U*_*a,j*_ is the apparent SED, and $f_{j}\left(x\right)=e^{-\frac{dist\left(x,j\right)}{D}}$ is the spatial influence function as proposed by Mullender et al. (Mullender et al., 1994). $dist\left(x,j\right)$ describes the distance between the *j* ^*th*^ element in the sum and the location of interest, x , whose local stimulus is being computed. The value *D* is the spatial influence parameter that equals the value needed to result in $f_{j}\left(D\right)/f_{j}\left(0\right)=e^{-1}$ .

**FIGURE 2:**
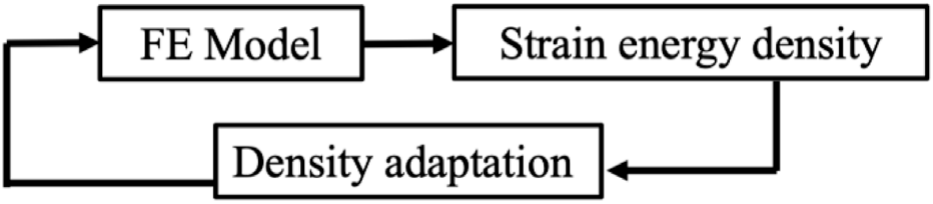
Feedback loop of adaptive FE.

The initial condition in all simulations was a homogeneous density 0.87 g/cm^3^, with a new density being calculated based on the SED condition after every remodeling iteration (Equation 1). Each element was linear, elastic, and isotropic with a Poisson’s ratio of 0.3 (Duchemin et al., 2008) and a Young’s Modulus being dependent upon the density (Table 1). A compressive stress was applied to the FE model that decreased linearly from 10 N/mm^2^ (left) to 0 N/mm^2^ (right) on the whole top face of the cube (Jang et al., 2009; Lian et al., 2010; Mullender et al., 1994; Rosenberg and Bull, 2018; Weinans et al., 1992), while the bottom face of the cube was fixed in the vertical direction (Figure 1). The results of the FE model informed the bone remodeling algorithm, which was called to calculate a new density; the new density was used to update the material properties of the FE model before rerunning the loading condition (Figure 2). The convergence criteria defined as meeting two conditions: the incremental density change, averaged over all the elements in the model, was below 0.01 g/cm^3^, and the simulation reached 200 iterations. This second criterion was added following preliminary simulations showing promising density evolutions early on but ending up in unsolvable structures for the FE software.

**TABLE 1:** Baseline parameters used in the test of the governing algorithm.

Parameter	Value	References
Initial density *ρ*_*o*_	0.87 g/cm^3^	Andreaus et al. (2012)
Density range *ρ*_*min*_ ≤ *ρ*_*j*_ ≤ *ρ*_*max*_	0.0174 ≤ *ρ*_*j*_ ≤ 1.74 g/cm^3^	Andreaus et al. (2012)
Spatial influence parameter *D*	0.20 mm	Moon et al. (2004); Liu et al. (2010)
Constant reference value *k*	0.025 J/g	Badilatti et al. (2016); Rosenberg and Bull (2018); Schulte et al. (2013)
Rate constant *τ*	1.0	Chen et al. (2007); Jang et al. (2009); Lian et al. (2010); Mullender et al. (1994)
Poisson’s ratio	0.3	Duchemin et al. (2008)
Young’s modulus	1904*ρ* ^1.64^ MPa	Wirtz et al. (2000)

To study the behavior of the SED-based bone remodeling algorithm combined with the spatial influence function, a sensitivity study was conducted. Three different relationships between Young’s modulus and density: *E*_*1*_ = 1904ρ^1.64^ MPa (Wirtz et al., 2000), *E*_*2*_ = 2038ρ^2.5^ MPa (Miller et al., 2002), and *E*_*3*_ = 3227ρ^3.0^ MPa (Andreaus et al., 2012), were implemented and investigated in this study. The spatial influence parameter *D* was varied around a baseline number of 0.20 mm; this value is similar to the finite-element length (Mullender et al., 1994; Rosenberg and Bull, 2018) and the length scale of trabecular thickness reported in imaging studies (Moon et al., 2004; Liu et al., 2010), resulting in *D* values of 0.10, 0.15, 0.20, 0.25, and 0.30 mm. The constant reference value *k* was varied: 0.005, 0.015, 0.025, 0.035, and 0.045 J/g, which represents values around the steady state condition of bone (Badilatti et al., 2016; Schulte et al., 2013) with a 1 g/cm^3^ bone-density assumption. Variation of rate constant $\tau =0.5,1.0,\text{and }2.0{\;\left(g/{\text{cm}}^{3}\right)}^{2}/\text{MPa}.\text{time unit}$ was also chosen around the established baseline value (Chen et al., 2007; Jang et al., 2009; Lian et al., 2010; Mullender et al., 1994). Details of the applied parameter values are summarized in Table 2 for all simulations.

**TABLE 2:** Details of parameter’s value in each simulation.

Sensitivity study parameter	Label	*D*	*k*	*τ*	*E*
Young’s modulus *E*	Baseline	0.20	0.025	1	*E_1_*
	S1	0.20	0.025	1	*E_2_*
	S2	0.20	0.025	1	*E_3_*
spatial influence parameter *D*	S3	0.10	0.025	1	*E_1_*
	S4	0.15	0.025	1	*E_1_*
	Baseline	0.20	0.025	1	*E_1_*
	S5	0.25	0.025	1	*E_1_*
	S6	0.30	0.025	1	*E_1_*
constant reference value *k*	S7	0.20	0.005	1	*E_1_*
	S8	0.20	0.015	1	*E_1_*
	Baseline	0.20	0.025	1	*E_1_*
	S9	0.20	0.035	1	*E_1_*
	S10	0.20	0.045	1	*E_1_*
rate constant *τ*	S11	0.20	0.025	0.5	*E_1_*
	Baseline	0.20	0.025	1	*E_1_*
	S12	0.20	0.025	2	*E_1_*

## Results

**FIGURE 3:**
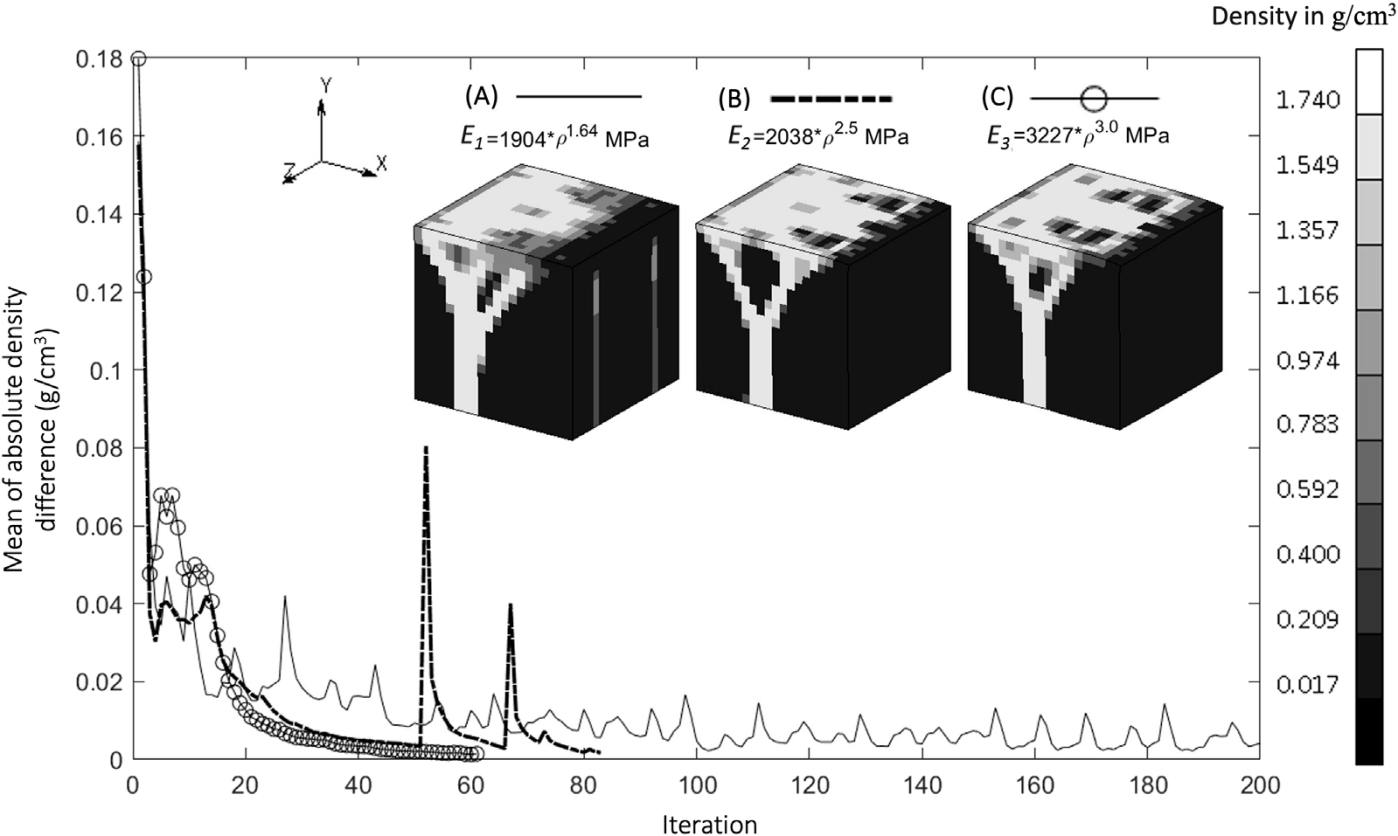
Convergence of mean of absolute density difference for three different Young’s Modulus relationships **(A)**. Baseline at iteration 200, **(B)**. S1 (higher power) at iteration 83, and **(C)**. S2 (highest power) at iteration 62 (Legend unit in g/cm^3^).

The density distributions for the baseline simulation with the three different Young’s modulus–density relationships are shown in Figure 3. The Young’s modulus relationships with higher powers, *E*_*2*_ and *E*_*3*_, resulted in more discrete areas than those with the lower power, *E*_*1*_. However, *E*_*1*_ provided a stable density distribution over a larger number of iterations, whereas *E*_*2*_ and *E*_*3*_ resulted in distributions unsolvable for the FE analysis before iteration 100. Therefore, the subsequent results used the *E*_*1*_ relationship and are presented after iteration 200 (unless otherwise specified), at which point the absolute average change of density between one iteration and the next reached below 0.01 g/cm^3^.

**FIGURE 4:**
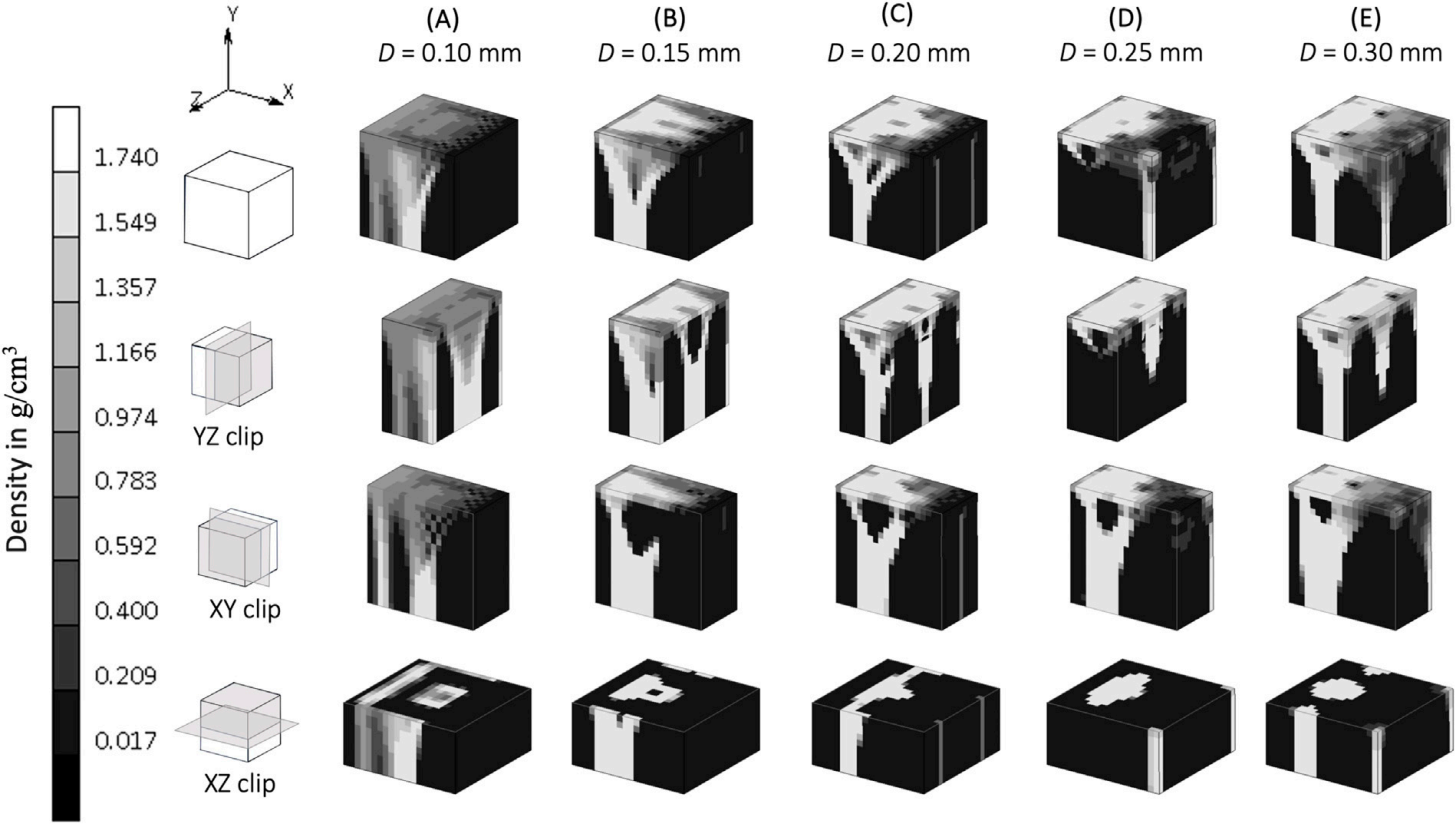
Sensitivity of bone trabecular shape to the spatial influence parameter *D* with increasing magnitudes from simulation **(A)**. S3, **(B)**. S4, **(C)**. Baseline, **(D)**. S5, and **(E)**. S6 at iteration 200. (Legend unit in *g/cm* ^*3*^).

The implementation of the SED-based spatial influence function in the 3D cube geometry for various spatial influence parameters is shown in Figure 4. For the sake of clarity and completeness, the entire part of the cube and several of its cross-sections are displayed. Higher values of *D* resulted in a reduction of the checkerboard pattern on density distributions. There were also variations in trabecular branching visible in all three sections (xy, yz, and xz), even though the load was only applied in the y direction.

**FIGURE 5:**
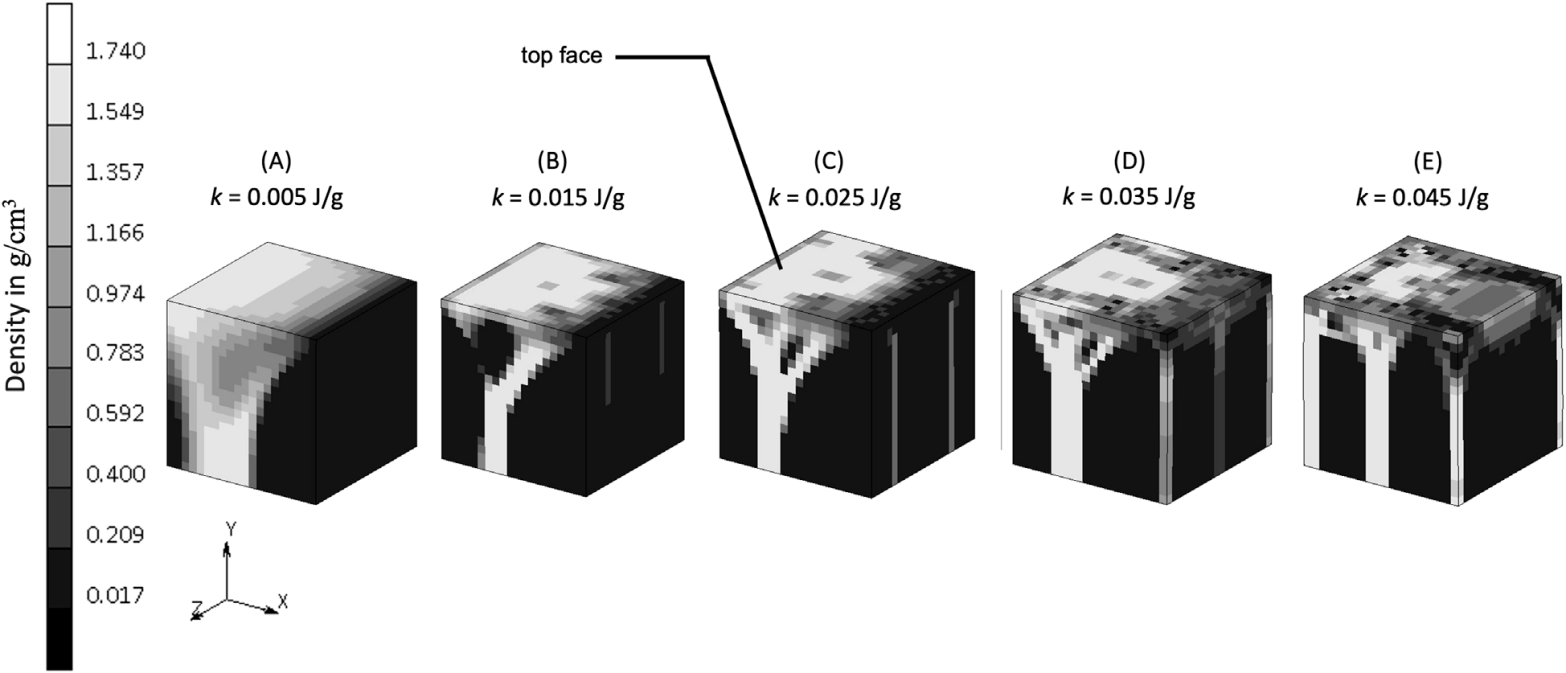
Sensitivity of bone trabecular shape to the reference value *k* with increasing magnitudes from simulation **(A)**. S7, **(B)**. S8, **(C)**. Baseline, **(D)**. S9, and **(E)**. S10 at iteration 200. (Legend unit is g/cm^3^).

The density distribution resulting from the remodeling algorithm is also sensitive to the constant reference value *k* (Figure 5). The final density distribution for the lowest *k* value shows thicker but blurrier struts than those with higher *k* value. The highest *k* value resulted in thin branches that also spread to the vertices of the geometry. As seen from the top face of each cube in Figure 5, the higher *k* value resulted in smaller trabecular struts than did the lower *k* value.

**FIGURE 6:**
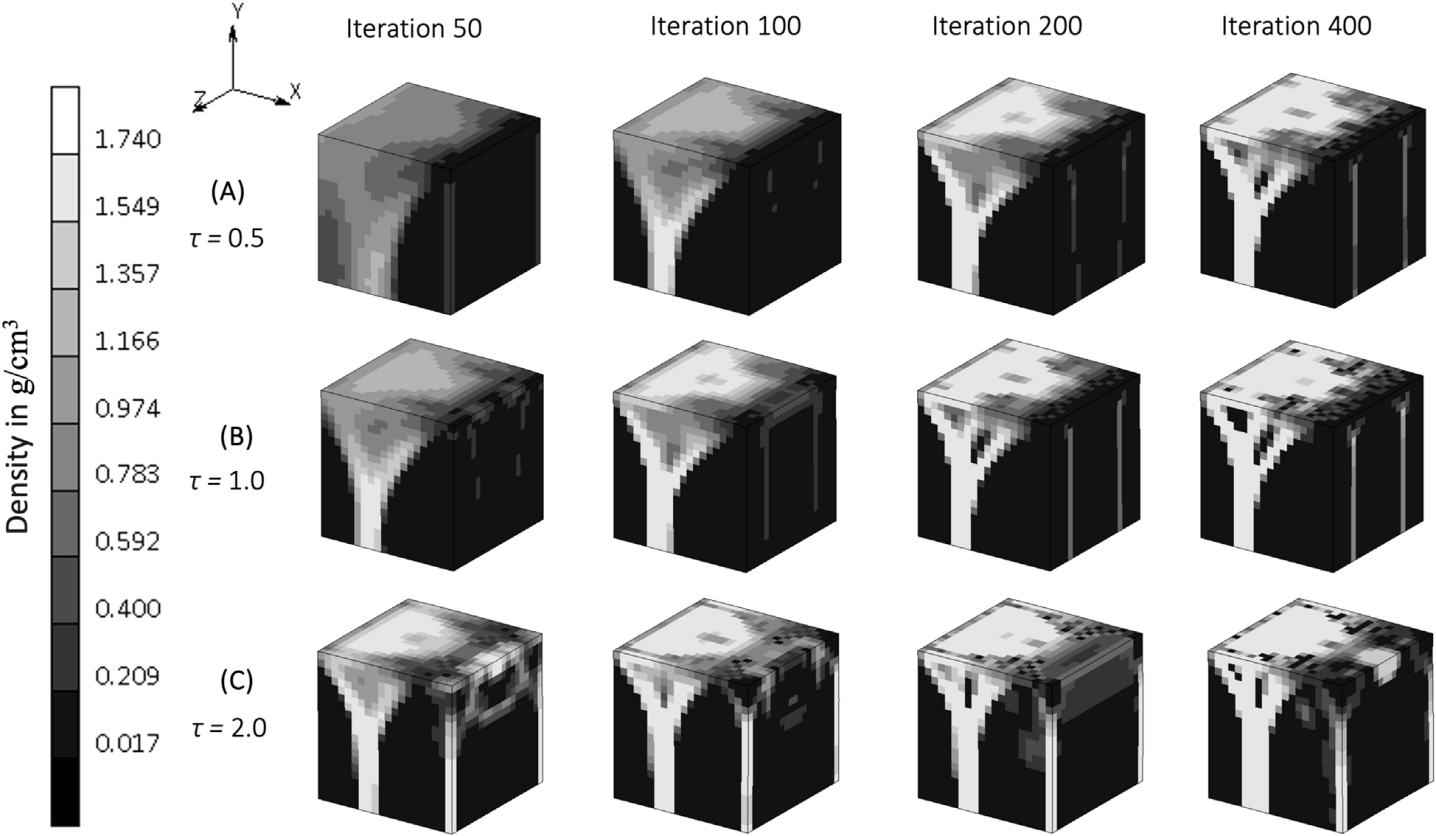
The evolution of density distribution resulting from various rate constants in ${\left(g/{\text{cm}}^{3}\right)}^{2}/\text{MPa}.\;\text{time unit}$ . **(A)**. τ=0.5 , **(B)**. τ=1.0 , and **(C)**. τ=2.0 . (Legend unit is g/cm^3^).

The rate constant, *τ*, in the bone remodeling algorithm impacts the evolution of the bone density distribution (Figure 6). Similar density distributions were reached in a shorter iteration time for a higher rate constant ( $1.0\;{\left(g/{cm}^{3}\right)}^{2}/MPa.dt$ ) compared to a lower rate constant ( $0.5{\left(g/{cm}^{3}\right)}^{2}/MPa.dt$ ). However, at the highest value (Figure 6C), the density distribution also concentrated at the corner of the geometry.

## Discussion

This is the first study to implement an SED-driven bone remodeling algorithm combined with a spatial influence function following Mullender’s work (Mullender et al., 1994), to create trabecular structures in simple 3D geometries. In comparison to 3D bone remodeling studies without a spatial influence function (Belinha et al., 2013; Shefelbine et al., 2005), this study enabled a density distribution that also propagated in directions not aligned with the external load. This study simulates the work of osteocytes in 3D, creating a non-local remodeling process due to spatial communication (Zhang et al., 2023). A sensitivity study was conducted on remodeling algorithm parameters, quantifying how different parameters influence not only bone mineral density but also the architecture of the resulting bone structure.

The relationship between Young’s modulus and density influences the resulting density distribution (Figure 3). At the same initial density, a higher power relationship results under stress in a higher Young’s modulus, which in turn creates a higher strain energy density (SED) and yields a higher density in the subsequent iteration. The dense region becomes denser with a higher power of Young’s modulus relationship, as indicated by a less blurry area (Figure 3C). These self-reinforcing mechanisms are at the core of the bone adaptation algorithm and are only exacerbated by higher power relationships between the Young’s modulus and the density, which could explain why the rule *E*_*1*_, with the lowest exponent, was the only one to convergence to a solution. Indeed, albeit seemingly displaying satisfactory density distributions (Figure 3), *E*_*2*_ and *E*_*3*_ resulted in “unstable” bone architectures which the FE solver could not reconciliate and could therefore not be considered to have achieved convergence. An important finding of this study is therefore that the presented bone remodeling algorithm is sensitive to the material law, making it a crucial design choice of such models. A higher power relationship (which means a higher value of Young’s modulus in this case) is suitable to represent cortical bone, or regions that consist mostly of cortical bone, such as in the mid-shaft of femoral bone [18 GPa (Cuppone et al., 2004)], which has a dense bone architecture (Suen et al., 2015). Lower values of Young’s modulus, such as in the trabecular bone of the femur [7.8 GPa (Mente and Lewis, 1989)], are associated with less dense bone architecture, shown as a blurry region in Figure 3A.

A spatial influence parameter *D* represents the power of influence delivered from one sensor to another sensor. Mullender et al. (1994) first introduced this as a distance from a sensor (which does not necessarily means the distance between osteocytes) at which its effect has reduced to 36.8 percent and assumed this influence domain has a similar order of magnitude than a trabeculae’s thickness, which varies from 0.20 to 0.40 mm (Moon et al., 2004; Liu et al., 2010). These values are the ones investigated in the sensitivity analysis of this study. A higher spatial influence parameter means that neighboring elements have a greater effect on the actual stimulus experienced by a particular region of the bone. This reduces the mesh dependency (Mullender et al., 1994) and mitigates numerical instabilities due to the checkerboard pattern (Gubaua et al., 2022; Mullender et al., 1994) as shown in the xy section of Figure 4. In agreement with Mullender et al. (1994), as long as the spatial influence parameter is higher than the element size, the checkerboard pattern is mitigated, and osteocyte function is able to be represented. Conversely, a lower value for *D* restricts the influence of the surrounding elements, which results in a model where the Mullender “sensing cell” assumption was absent altogether (Figure 4), with the density distribution only following the direction of the external load.

**FIGURE 7:**
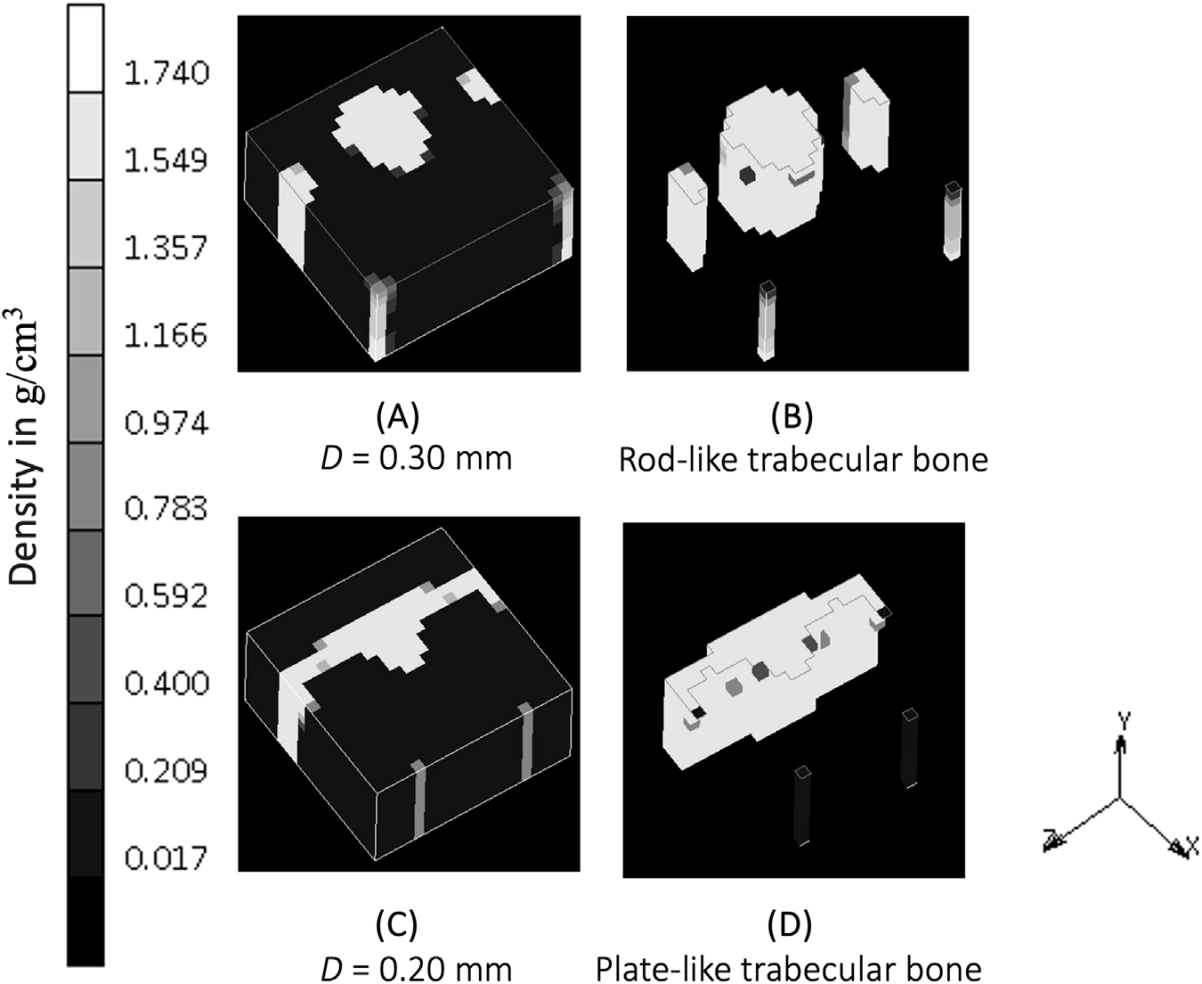
**(A)** Density distribution after simulation S6 (D = 0.30 mm) at iteration 400. **(B)** Plot clipping the result of simulation S6 showing rod-like bone architecture. **(C)** Density distribution after baseline simulation (D = 0.20 mm) at iteration 400. **(D)** Plot clipping the result of baseline simulation showing plate-like bone architecture.

This study shows that when the “sensing cell” assumption is applied, which is represented by a higher value of spatial influence parameter, there is a density distribution that also “propagates” in a direction not directly aligned with the external load (xz and yz section of Figure 4). Studies that did not implement the spatial influence concept (Belinha et al., 2013; Shefelbine et al., 2005) were unable to show the interaction of osteocytes, and resulted in the direction of the density distribution being only dependent on the direction of the external loading. The spatial influence function also enables the architecture type of trabecular bone to be mimicked. Trabecular bone has been classified into plate-like bone (flatter bone architecture) and rod-like bone (elongated and cylindrical bone architecture) (Salmon et al., 2015). “Plate-like” density architecture is shown in the xz section of the baseline simulation (*D* = 0.20 mm), meanwhile the “rod-like” density is shown in S6 (*D* = 0.30 mm) in the same xz section. Different spatial influence parameter values may, therefore, be applied to different sites. For example, the *D* = 0.20 mm scenario (if a fine mesh is feasible) that represents “plate-like” bone is likely suitable for simulating the detailed view of the femoral head (Figure 7), as the structure of femoral head observed by micro-CT imaging is characterized by plates (Hildebrand et al., 1999; Issever et al., 2003; Kothari et al., 1998) and has average trabecular thickness of 0.194 mm (Hildebrand et al., 1999). For larger domain FE applications, the value of *D* is suggested to be similar to the element length, ensuring that the stimulus is neither under- nor over-influenced. The effect of the spatial influence parameter on trabecular structure illustrates how changes in bone sensing, likely mediated by cellular signaling and biological processes, can occur even in the absence of changes in the loading environment. Felder et al. (2021) measured bone architecture with the same local sensing (at the same site) but under different loading conditions. An Ellipsoid Factor (EF) was utilised to characterize the plate-to-rod transition in trabecular bone at the proximal tibia under conditions of reduced loading (disuse osteoporosis). The results support the notion that the plate-to-rod transition does not coincide with the onset of bone loss.

**FIGURE 8:**
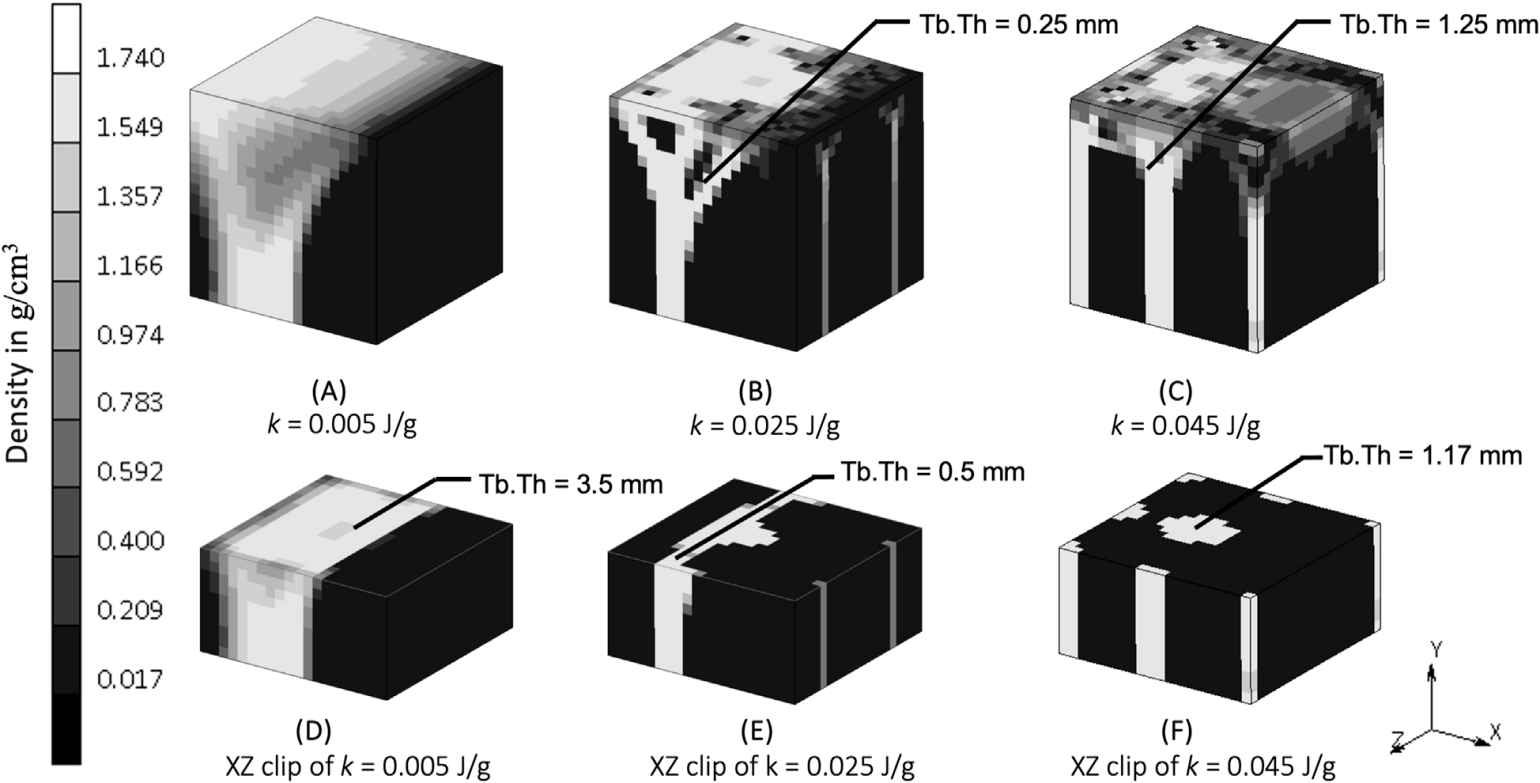
Density distribution after simulation **(A)** S7 (*k* = 0.005 J/g), **(B)** baseline (*k* = 0.025 J/g), and **(C)** S10 (*k* = 0.045 J/g). Sectional view at y = 2.5 mm of simulation **(D)** S7, **(E)** baseline, and **(F)** S10. Tb.Th is trabecular thickness.

The constant reference value represents a balance condition where the process of bone formation and resorption are in equilibrium. The lower *k* allowed more bone formation and less resorption to occur, as could be expected, resulting in thicker bony structures (Figure 5A). Increasing *k* allowed less bone formation and more resorption to occur, resulting in a thinner structure (Figure 5E), that led to a decrease of mass in the system (Lian et al., 2011; Su et al., 2019). This is similar to the microstructure of post-menopausal bone (Badilatti et al., 2016; Mueller and Hayes, 1997), which exemplifies how adjusting the model’s mechanobiological parameters can help reproducing the physiological imbalance between bone formation and resorption seen in real-life scenarios. Setting the level of *k* is important, and although a value of 0.004 J/g has been used previously (Weinans et al., 1992; Mellal et al., 2004; Zhang et al., 2016; Prochor and Sajewicz, 2018), this has not been shown to lead to realistic trabecular bone configuration in 3D. The result from the sensitivity analysis suggests that a value of *k* at the order of 0.025 J/g results in adequate thickness across all branches (see the middle column of Figure 8), making it suitable for representing trabecular bone in 3D simulations. This is supported by CT imaging data showing trabecular thickness varying from 0.2 to 0.4 mm (Moon et al., 2004; Liu et al., 2010), and being as large as 0.7 mm in distal forearm specimens (Issever et al., 2010), depending on bone location and function.

The rate constant, *τ*, influences the rate of bone formation and resorption, representing the work done by osteoblasts and osteoclasts *in vivo.* The evolution of bone density in the simulations, reaching the same value at different iterations depending on the rate constant is demonstrated clearly in Figure 5. This “delayed similarity” was more evident between the lower two implemented rates (between iterations 100, 200 and 400 of *τ* = 0.5 and iterations 50, 100 and 200 of *τ* = 1) than with the higher ones (*τ* = 2), suggesting a non-linear relationship between the rate of bone remodeling and the resulting density distributions, perhaps indicative of different evolutionary patterns. If bone adapts too quickly, it may not have enough time to balance properly, potentially leading to damage, fractures, overgrowth, or structural instability, consistent with the more disorganized and chaotic structure seen in Figure 5C. A rate constant of 1 [(g/cm^3^)^2^/MPa. time unit] has been used in previous studies to represent heterotopic ossification in cervical total disc replacement (Ganbat et al., 2016), bone changes in dental implants (Mellal et al., 2004), and osseointegration in posterior lumbar interbody fusion (Zhang et al., 2016) and in the femur when using an osseointegration prosthesis (Prochor and Sajewicz, 2018). Although this value for the rate constant appears to be accepted, different rate constants might be appropriate to represent bone remodeling differences with age.

Future work could investigate the influence of mesh size in trabecular structure, as this study did not include a mesh-sensitivity analysis, despite the spatial influence function being commonly cited for mitigating mesh dependency. Additionally, the study only considers a single load case at the macroscopic, 5 mm cube scale, even though various physiological loading scenarios are possible. It was deemed appropriate to start by carrying out an extensive analysis of a basic case under one loading condition. Similar to other remodeling publications (Mullender et al., 1994; Rosenberg and Bull, 2018) the findings of which suggest that the learnings translate to more complex situations, the results from the work presented here suggests that the algorithm has the potential to be adapted to simulate successfully additional loading conditions and geometries.

This methodology can be feasibly scaled up for larger domains, such as long bones. To address the computational expense, future studies could apply fine meshing selectively in regions of interest to ensure accurate representation of trabecular thickness. Additionally, this approach can be extended to non-Cartesian mesh models, such as 3D adaptive tetrahedral meshes, as stimuli measure strain energy density at the centroid integration points of elements, with spatial influence values representing distances between neighboring elements. Subroutines in finite element software can be utilised to calculate these distances between integration points.

This study has implemented an SED-based bone remodeling algorithm with a spatial influence function in simple 3D geometries and shown to produce sensible bone structures. Key model parameters were shown to affect the resulting bone structures, and so, depending on application, appropriate parameters should be chosen to reflect the physiological conditions modelled.

## Data Availability

The original contributions presented in the study are included in the article/supplementary material, further inquiries can be directed to the corresponding author.
